# Preventing Neurons and Glial Cells Destruction in Substantia Nigra Pars Compacta and Striatum Using St. John's Wort and Stem Cells on Parkinson's Model

**DOI:** 10.1002/brb3.71124

**Published:** 2025-12-07

**Authors:** Hamed Farzadmanesh, Hamidreza Sameni, Ali Ghanbari, Abbas Ali Vafaei, Ali Khaleghian, Majid Mirmohammadkhani, Laya Ghahari, Afsaneh Shokri, Manouchehr Safari

**Affiliations:** ^1^ Nervous System Stem Cells Research Center Semnan University of Medical Sciences Semnan Iran; ^2^ Research Center and Department of Physiology Semnan University of Medical Sciences Semnan Iran; ^3^ Cancer Research Center Semnan University of Medical Sciences Semnan Iran; ^4^ Social Determinants of Health Research Center Semnan University of Medical Sciences Semnan Iran; ^5^ Department of Anatomy AJA University of Medical Sciences Tehran Iran; ^6^ Department of Foundation Studies Global College of Engineering and Technology Muscat Sultanate of Oman

**Keywords:** 1‐methyl‐4‐phenyl‐1,2,3,6‐tetrahydropyridine (MPTP), Parkinson's disease, St. John's wort, stem cells, striatum, substantia nigra pars compacta

## Abstract

**Study Objective:**

St. John's wort (*Hypericum perforatum*) has been used for centuries as a medicinal plant to remedy external disorders such as burns and wounds, as well as internal disorders such as nerve pain, anxiety, and depression. Bone marrow mesenchymal stem cells (BMSCs) are a heterogeneous population of pluripotent stromal cells that can differentiate into a variety of cell types. The aim of the study was to individually and combinationally investigate the effect of whole extract and stem cells on Parkinson's disease. Furthermore, using a new method for stem cells transplantation to enhance cell integration into substantia nigra pars compacta (SNC) and striatum.

**Methods:**

In the study, 63 adult male Wistar rats were classified, and among them, treatment groups received 20 mg/kg extract (intraperitoneally; 28 days) and nearly 1 million (mean of 936 × 10^3^) mesenchymal stem cells through cisterna magna, either individually or in combination.

**Results:**

Histological evaluations revealed that there has been a sharp rise in neurons in both ventral and dorsal striatum in the pretreatment plus cell group (*p *< 0.001). Regarding neurons in SNC area, quantitative investigations via Nissl and immunohistochemistry staining showed significant differences between the lesion group and groups receiving pretreatment, treatment plus cells, and pretreatment plus cells (*p *< 0.001). According to evidence, there are some communications between St. John's wort and stem cells that have led to navigate stem cells to immigrate and deploy within striatum.

**Conclusion:**

The study found that both *H. perforatum* extract and BMSCs can help preserve dopaminergic neurons and glial cells during which combination therapy demonstrates synergy as an indicator. St. John's is a natural preserver and appears to function as a neuroprotective agent and facilitate the navigation and integration of stem cells; consequently, it keeps surviving both neurons and glial cells against pathogens and destructive environmental factors and has a potential to help us and Parkinson's patients.

## Graphical abstract

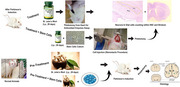

**Abbreviations**
6‐OHDA: 6‐hydroxydopamine
AP: anteroposterior
bFGF: basic fibroblast growth factor
BMSCs: bone marrow mesenchymal stem cells
CD90: cluster of differentiation 90
CNTF: ciliary neurotrophic factor
DiI: 1,1′‐dioctadecyl‐3,3,3′,3′‐tetramethylindocarbocyanine perchlorate
DV: dorsoventral
FBS: fetal bovine serum
i.p.: intraperitoneal
ML: mediolateral
MPTP: 1‐methyl‐4‐phenyl‐1,2,3,6‐tetrahydropyridine
PBS: phosphate buffered saline
PD: Parkinson's disease
Pen‐Strep: penicillin‐streptomycin solution
ROS: reactive oxygen species
SHP: standardized *Hypericum perforatum* extract
SNC: substantia nigra pars compacta
TH: tyrosine hydroxylase

## 1 Introduction

Parkinson's disease (PD) is a progressive and chronic disorder of the central nervous system that is characterized by extensive changes, including non‐motor symptoms such as boredom, sleep, smell, cognition, dementia, and hallucinations and also motor symptoms like head tremor, muscle stiffness, postural instability, and difficulty walking, among others. One of the main causes of PD is the degeneration of dopaminergic neurons in the substantia nigra pars compacta (SNC), leading to dopamine depletion in the striatum and impairing in motor control (Cerasa et al. 2016). Although the exact cause of this degeneration remains unknown, current remedies focus on symptom management rather than halting disease progression. Mesenchymal stem cells can be isolated from different tissues, that is, both embryonic and adult tissues which include skin, peripheral blood, fat, umbilical cord blood, Wharton's jelly, and bone marrow. Bone marrow mesenchymal stem cells (BMSCs) are multipotent cells that can differentiate into a variety of cells. Research has shown that BMSCs have neuroprotective effects via reducing apoptosis and neural death, and they can also protect tyrosine hydroxylase (TH)^+^ cells in the SNC of the brainstem (He et al. 2019; Suzuki et al. 2015).

St. John's wort (*Hypericum perforatum*) is a native European plant that is also found in the United States, Western Asia, and the north of Africa. St. John's wort has been used for centuries as an herbal medicine for the treatment of external disorders such as burns and ulcers, and also internal disorders such as neuropathic pains and depression (Galeotti 2017). The major components of St. John's wort include quercetin, hyperoside, quercitrin, rutin, hypericin, kaempferol, biapigenin, and hyperforin (Oliveira et al. 2016). The concentrations of major bioactive compounds in St. John's wort vary depending on factors such as harvesting time, temperature, and germplasm. For instance, Couceiro et al. (2006) investigated the effect of two different temperatures and found that hyperforin concentration remained higher at 25°C than at 30°C. All of these components have their own unique properties; for example, neuroprotective effects of quercetin against H_2_O_2_‐dependent apoptosis in *SH‐SY5Y* human neurons were investigated, and it was observed to stop the caspase cascade and prevent DNA fragmentation (Suematsu et al. 2011). Similar results were observed in which hyperoside by stopping caspase enzymes prevents apoptosis (Zeng et al. 2011). Hypericin and hyperforin are also well known for their neuroprotective properties. These substances have been demonstrated to affect various cellular processes associated with neurodegeneration. They can operate as antioxidants, anti‐inflammatory agents, and regulators of neurotransmitter systems, potentially helping to slow the progression of neurodegenerative diseases (Suryawanshi et al. 2024). As St. John's and its ingredients like quercetin have potential in neurogenesis and enhance the maturation of neural progenitor into neurons in the dentate gyrus of the Alzheimer's disease model, exploring their neuroprotective effects on PD might be useful (Karimipour et al. 2019). Kaempferol has also exhibited antiapoptotic and antioxidant properties, as it severely protects mitochondrial network about transmembrane transmission and oxygen consumption and reduces mitochondrial reactive oxygen species (ROS) and carbonyl levels (Filomeni et al. 2012). All these studies proved that St. John's wort could have a potential for recovery or preventing PD. So, the prevention of disease progression or onset might be connected with the St. John's consumption which is a famous medicinal plant in the West like Borage (*Echium*) in the Middle East and the Far East. Selecting combined treatment is a purpose to determine whether the medicine can direct stem cells to reach and integrate into the SNC and striatum. Furthermore, testing individual ingredients of St. John's wort in Parkinson's models using effective dosages and novel delivery methods such as gene therapy—especially with compounds like kaempferol, hyperforin, quercetin, or rutin—is recommended for future research.

## 2 Materials and Methods

### 2.1 Animals and Model

This research was done on 63 male Wistar rats weighing 200–300 g. Animals were kept in the animal house of SEMUMS in a 12 h light/dark cycle in transparent cages at an ambient temperature of 22–25°C individually. Water and food were also available ad libitum. The protocol number and certificate of animal ethics for this research were issued from Semnan University of Medical Sciences Ethics Committee which is IR.SEMUMS.REC.1396.188 with sub‐number 1328 to do the research.

To create a Parkinson's model, we used 1‐methyl‐4‐phenyl‐1,2,3,6‐tetrahydropyridine (MPTP) hydrochloride (Sigma) which can be administered in various ways, including injections into the SNC, intraperitoneal (i.p.), subcutaneous, and intranasal. Multiple clinical evidences have shown that the intranasal injection of MPTP is very efficient for the induction of Parkinson's via olfactory tract (Ayton et al. 2013; Cannon and Greenamyre 2010; Nonnekes et al. 2018). In this study, we consumed a single bilateral dose of the neurotoxin intranasally, so we first dissolved MPTP in ethanol (10% w/v) and saline (0.9% NaCl) at a concentration of 2 mg/mL to prepare a stock solution. After that, we anesthetized the rats using ketamine/xylazine (80/10 mg/kg) and inserted then a pre‐marked PE‐10 microtube into the nostril (10 mm) and connected microtube to an electrical pump (WPI, syringe pump, SP101iz). Next, we performed one bilateral injection (0.1 mg/nostril) with a flow rate of 12.5 µL/min over 4 min (Castro et al. 2013; Prediger et al. 2006; Soares et al. 2025). A week after injections, Parkinson's animals were confirmed by performing the pole test.

### 2.2 Pole Test

Pole test is a locomotor activity test that is used for evaluation of bradykinesia 1 week after MPTP injection. This method was first designed by Ogawa et al. Related apparatus includes a vertical column with a height of 100 cm and a diameter of 2.5 cm, with a 3 cm diameter pole that is fixed at the top and overall positioned inside a cage. The position of the rat should be head‐upward at the summit of the pole. The total time was evaluated to compare and includes “time to turn” and “time to descend.” The “time to turn” refers to the rat turning its head from an upward to downward position, and “time to descend” refers to descending from the pole to the floor (Ogata et al. 2003). Rats were tested after three training trials.

### 2.3 Experimental Design

Rats were randomly divided into nine groups of seven which are explained in the following:

**Group1** (control): It was an ordinary, healthy group. **Group2** (lesion) (L): This group received MPTP intranasally and did not receive any medicinal cure. **Group3** (MPTP vehicle): This group received normal saline and 10% ethanol through the nostrils. **Group4** (stem cells): This group received approximately 936 × 10^3^ mesenchymal stem cells (by 20 µL culture media) via cisterna magna 1 week after Parkinson's induction and performing the pole test (Kim et al. 2010). **Group5** (stem cells vehicle): This group received 20 µL culture media through cisterna magna. **Group6** (pretreatment + L): This group was pretreated with standardized *H. perforatum* extract (SHP) at a dose of 20 mg/kg/day for 4 weeks (i.p.) (El‐Sherbiny et al. 2003; Sanchez‐Reus et al. 2007), but they did not receive any treatment after Parkinson's induction. **Group7** (L + treatment): This group was treated with the extract at a dose of 20 mg/kg/day for 4 weeks (i.p.). **Group8** (L + treatment + cell): The group was treated with both extract (20 mg/kg/day; 4 weeks) and stem cell (nearly 1 million) via i.p. and cisterna magna, respectively. **Group9** (pretreatment + cell + L): The group was pretreated with extract at the same dose (4 weeks; i.p.) and received stem cells via cisterna magna.

### 2.4 Stem Cells Study

#### 2.4.1 Cell Culture

The source of our stem cells was rat's tibia and femur bones. You need some materials for cell culture; the required materials are listed below: Culture Media (DMEM/F12; Millipore, USA); fetal bovine serum (FBS) (Cat.10270‐106; Bioidea, Iran); Pen‐Strep (Cat.P06‐07100; PAN‐Biotech, Germany); Trypsin‐EDTA (0.25%; Lot.1881755; GIBCO). The stages are as follows: (1) extracting the stem cells, (2) centrifuging at 1200 RPM for 10 min, (3) adding 2 cc of culture media to obtain a uniform solution, (4) transferring the cell suspension into the flask and adding 3 cc of culture media, 0.5 cc of FBS and 50 µL of Pen‐Strep, and (5) placing the flask in a Co_2_ incubator at 37°C with 5% Co_2_ (Zarbakhsh et al. 2019).

#### 2.4.2 Flow Cytometry

Centrifuging cultured cells (1500 rpm; 5 min) and bringing the cell precipitation to the final volume by 1 mL phosphate buffered saline (PBS), then selecting 10 flow cytometry tubes and adding 100 µL cell suspension to each one. The antibodies used were as follows: (1) monoclonal antibody, (2) IgG1‐FITC, isotype control (5 µL); (3) IgG1‐PE, isotype control (5 µL); (4) IgG1‐Percp5.Cy5.5, isotype control (5 µL); (5) cluster of differentiation 90 (CD90)‐Percp, anti‐rat BioLegend (5 µL); (6) CD44‐FITC (5 µL); (7) CD34‐PE (5 µL); (8) CD45‐FITC (5 µL); (9) they were shaken and incubated at 4°C for 30 min, after which 500 µL of PBS was added; (10) centrifuging at 1500 rpm (5 min); finally adding 250 µL of the PBS buffer to the cell precipitation and analyzing the samples using a BD FACS Calibur flow cytometer (USA) (Cavaglieri et al. 2009). Recommended usage amount of antibody (10^6^ cells/100 µL): CD45 and CD90, ≤0.25 µg; CD34, ≤1.0 µg; CD44, ≤0.5 µg.

#### 2.4.3 Cells Transplantation and Tracing Them

Shutting the animal into the stereotactic device and perforating skull in front of the occipital crest on the midline at the following coordinates relative to bregma (−15.72 mm anteroposterior [AP]; 0 mm ML; 6–8 mm dorsoventral [DV] below the skull surface). Using a PE‐10 polyethylene catheter, we pierced the dura mater and advanced toward the cisterna magna from the posterior side of the cerebellum (J. S. Lee et al. 2015). To control the depth of entry, an 8 mm stopper was affixed to one end of the catheter. We extracted 20 µL of rat CSF to equalize pressure, then injected the stem cells with 20 µL of culture media using a Hamilton syringe and catheter, and finally sealed the skull opening with a screw. CellTracker CM‐DiI (Invitrogen) was used to trace the cells and determine whether the injected cells from the cisterna magna migrated to the striatum and SNC. This procedure was performed on one rat from each cell‐recipient group due to DiI's potential toxicity (cells were incubated with DiI at 37°C for 30 min) (Li et al. 2008). This method of injection is invasive but efficient. Other research has confirmed the long‐term safety of cisterna magna injections, and the technique is being further developed and investigated (Güresir et al. 2015; Park et al. 2022; Yoon et al. 2016).

### 2.5 Histopathology

#### 2.5.1 Nissl Stain

All sections were deparaffinized and hydrated using xylene followed by a graded alcohol series, respectively. Sections were stained in Cresyl Violet Acetate (1%) in a 65°С incubator for 2 min and then washed in two jars of distilled water for 5 min. They were placed into the acid alcohol solution (0.25% acetic acid, 50% ethanol; 3–5 sits) to remove excess color and washed with distilled water again. The slides were dehydrated in 96% and 100% ethanol and cleared by xylene. A cover‐glass was laid, and dorsal and ventral parts of the striatum were examined microscopically (Mansour et al. 2018).

#### 2.5.2 Immunohistochemistry (IHC)

**First**: Sections were incubated in citrate buffer (90°C; 15 min). **Second**: Endogenous peroxidase activity was blocked (3% H_2_O_2_ and 10% methanol; RT; 10 min). **Third**: Blocking nonspecific antigens (TBS; containing 0.3% Triton‐X100 and 10% FBS; RT; 2 h). **Fourth**: Slides were dried using filter paper. **Fifth**: Primary antibody (anti‐TH antibody, rabbit IgG, monoclonal, 1:750 in PBS with 5% FBS; Abcam) was applied and incubated overnight in a dark, humid chamber at room temperature. **Sixth**: Washed with TBS. **Seventh**: Secondary antibody (anti‐mouse/anti‐rabbit IgG, 1:1000 in PBS with 5% FBS; RT; 2 h; HRP type; BIOCYC GmbH & Co KG). **Eighth**: Using DAB chromogen (50 mg Diaminobenzidine, 100 mL Tris, and 0.03% H_2_O_2_), 2–3 drops for 10 min. **Ninth**: Sections were counterstained with hematoxylin and mounted (Zhao et al. 2016).

#### 2.5.3 Cell Count Strategy

**FIGURE 1:**
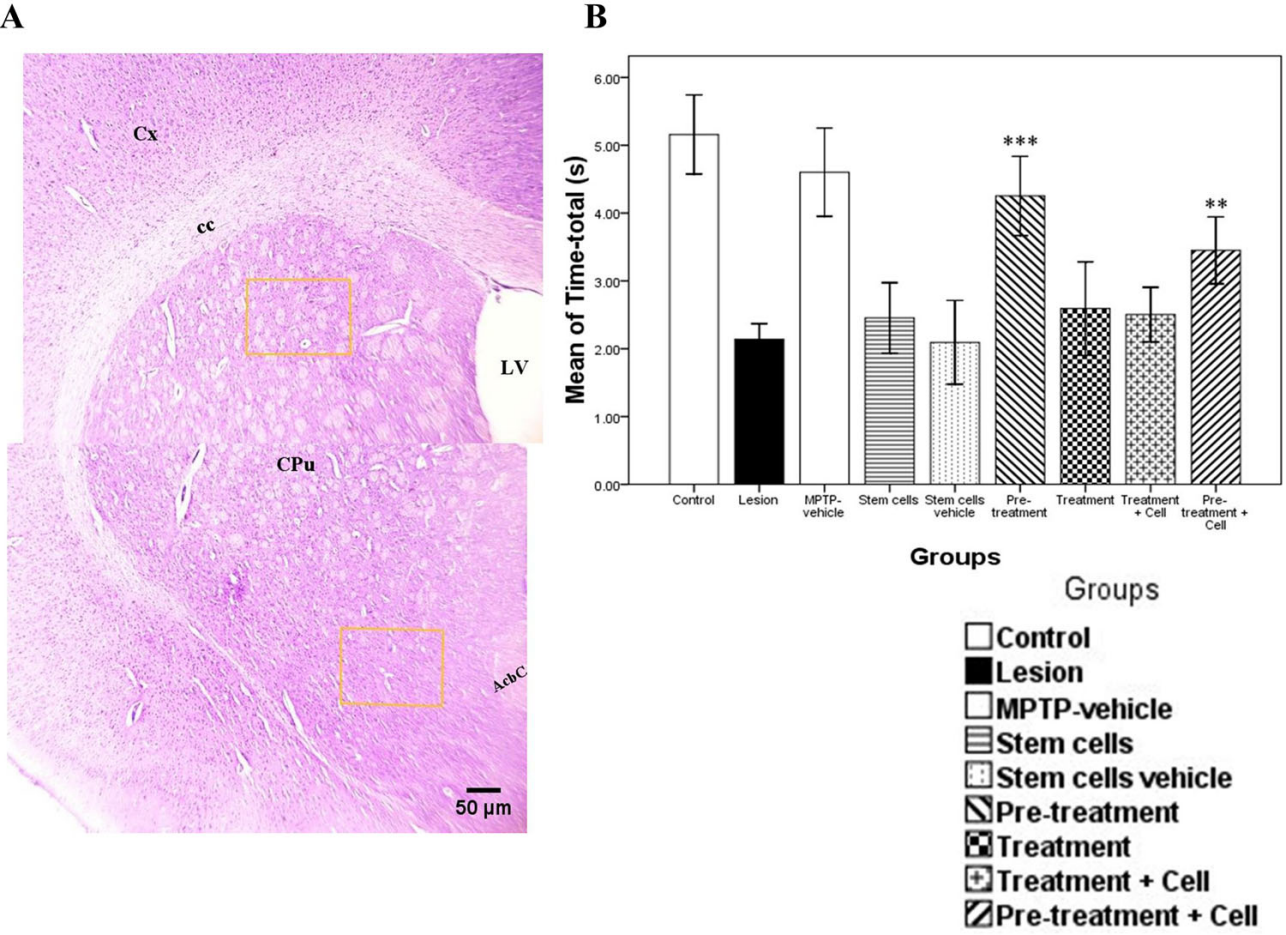
The location of the evaluated area in the ventral and dorsal parts of the striatum (caudate putamen) that is indicated by brown square. Based on the terms included in the Paxinus Atlas: Cx, cerebral cortex; cc, corpus callosum; CPu, caudate putamen (striatum); LV, lateral ventricle; AcbC, accumbens nucleus, core. Magnification ×40; scale bar 50 µm; Nissl stain (A). Data are expressed as the mean ± SD of time‐total (s) for pole test and were analyzed using one‐way ANOVA followed by the Tukey post hoc test; **p < 0.01; ***p < 0.001 (B). MPTP, 1‐methyl‐4‐phenyl‐1,2,3,6‐tetrahydropyridine.

By means of the Nissl staining in striatum or caudate‐putamen (CPu) and SNC, neurons and glial cells were identified and counted on the basis of criteria such as nucleus size and shape. Neurons typically have large size with round, oval, or polygonal euchromatic nuclei shape, whereas glial cells have small size with round or occasionally oval hyperchromatic nuclei (Diguet et al. 2004). The IHC stain was performed exclusively for dopaminergic neurons. Cell counts were performed in both ventral and dorsal parts of the striatum in a rectangular area with dimensions of 611 µm length and 455 µm width, covering an area of approximately 278,005 µm^2^ or 278 mm^2^ (Figure 1A). The sections from the brain and midbrain (mesencephalon) were cut and stained according to the Paxinos atlas with coordinates of interaural 4.6–2.3 mm for the striatum and 10.56–9.48 mm for the SNC area, according to AP orientation in the Paxinos atlas.

### 2.6 ELISA

The activity of antioxidant enzymes was measured such as superoxide dismutase (SOD) and glutathione peroxidase (GPX) in rat's blood serum using the Multi‐Mode Microplate Reader device (Synergy H1MFD; BioTek, USA) during which desired blood was taken from the heart. The SOD and GPX assay kits (Lot.ZB‐A518122; Lot.ZB‐A718220; ZellBio, Germany) were used, and the activities were determined colorimetrically on the basis of the kit formula (Pardo‐Andreu et al. 2008; Rostami et al. 2020).

### 2.7 Statistical Analysis

Values are expressed as mean ± SD and were analyzed using one‐way analysis of variance (one‐way ANOVA) followed by the Tukey post hoc test (*n* = 5–7). The SPSS software was used (version 16), and *p *< 0.05 was considered significant statistically. Error bars represent the 95% confidence interval.

## 3 Results

### 3.1 Pole Test Assay

There was a significant difference between control and MPTP vehicle with the lesion group (*p *< 0.001). There were no significant differences between the stem cells, stem cells vehicle, treatment, and treatment plus cell groups and the lesion group (*p *> 0.05), as this test was conducted before therapeutic intervention, confirming the induction of PD. However, a significant difference was observed between the pretreatment group (*p *< 0.001) and the pretreatment plus cell group (*p *= 0.003) compared to the lesion group, as this test was conducted after the intervention (Figure 1B).

### 3.2 Cell Culture Status and DiI Tracing

**FIGURE 2:**
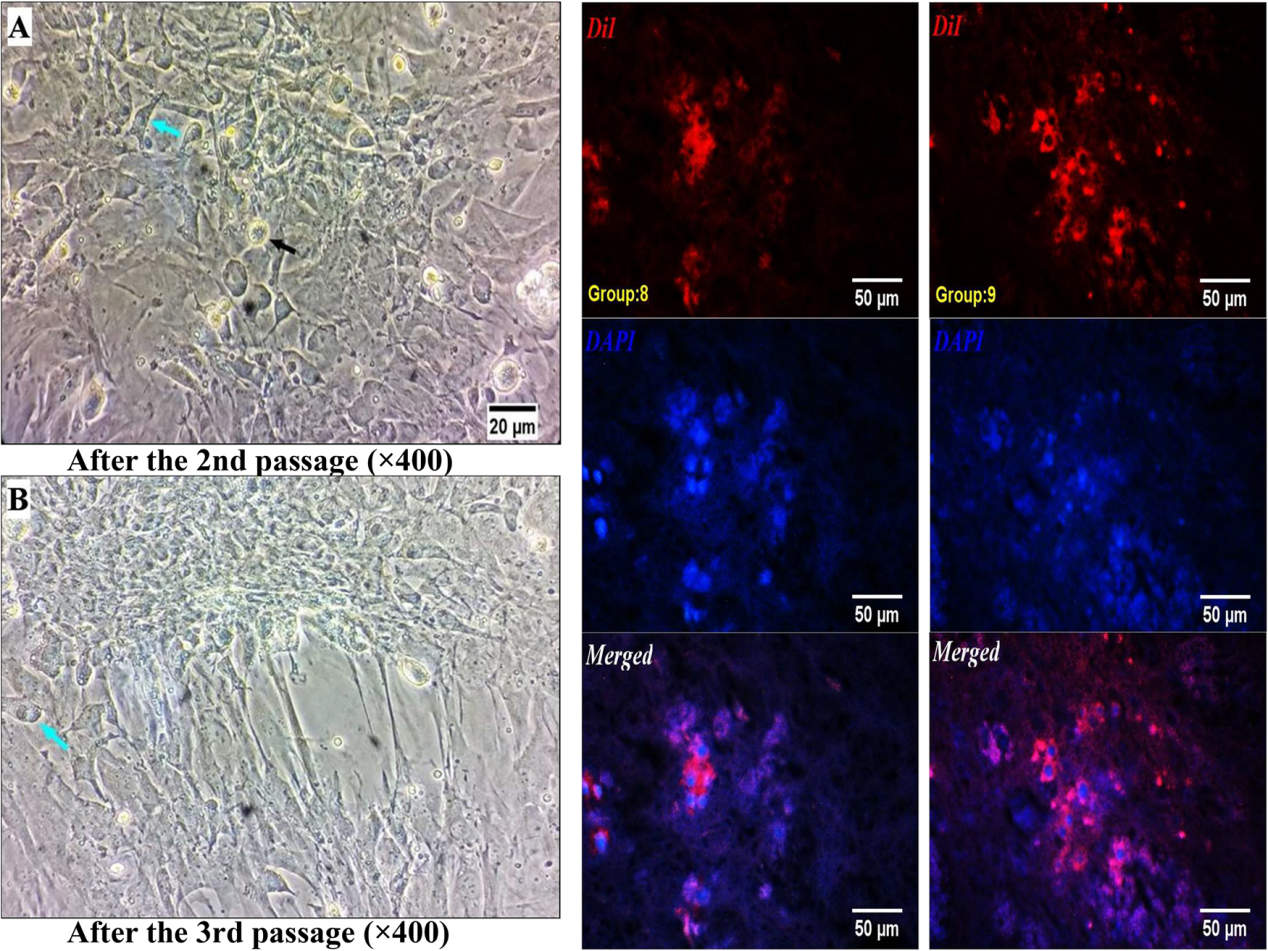
On your left: The inverted microscope images of stem cells after the second (A) and third (B) passages. Magnification ×400; scale bar 20 µm; cyan arrow indicates an alive cell, and black arrow indicates a dying cell. On your right: The inverted fluorescence microscope images of the striatum regarding cell tracking within the two groups of treatment plus stem cells (Group 8) and pretreatment plus stem cells (Group 9) that are arranged in two columns. Magnification ×400; scale bar 50 µm; DiI staining.

Evaluation after stem cell extraction showed that the cells were spherical, varied in size, and included stromal cells, red blood cells, fat cells, and fibroblasts. The culture medium was changed 48 h after cell extraction. By the fourth day, more spindle‐shaped cells with long appendages appeared, along with the formation of several cell colonies. By Day 9, stromal cells had covered over 90% of the confluence, and the number of colonies had significantly increased. The first cell passage was performed at this point. In both the second and third passages, the cells continued to take on a spindle shape and adhered to the flask, demonstrating a high proliferation rate. Evaluations confirmed the presence of stem cells in the striatum, and they were likely transported via cerebrospinal fluid from the cisterna magna to the fourth ventricle, then to the lateral ventricles, and finally into the striatum (Figure 2). However, there was no evidence of stem cell presence in the SNC.

### 3.3 Flow Cytometry Results

**FIGURE 3:**
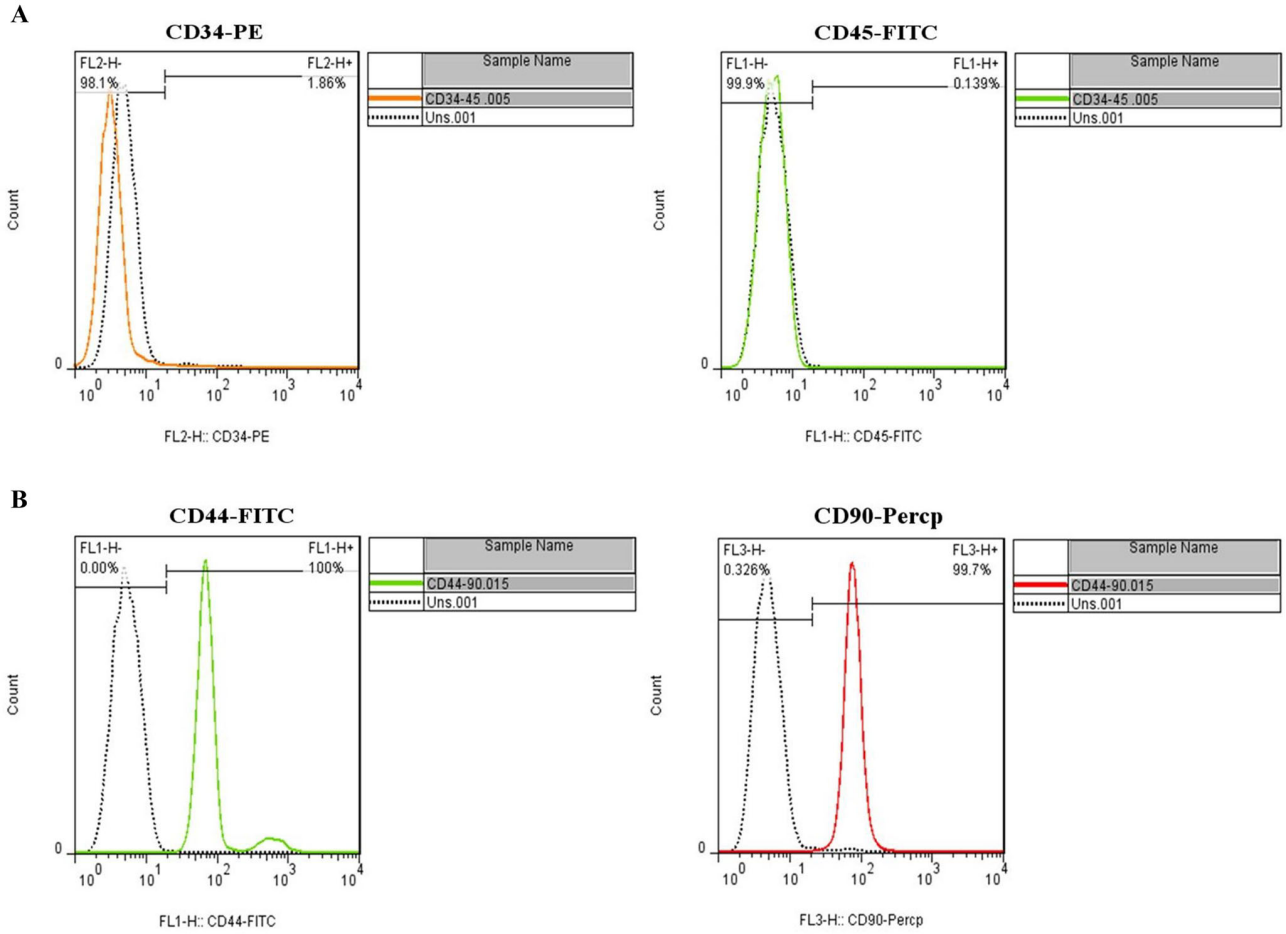
The expression of the cell surface markers related to BMSCs was measured by flow cytometry. Both CD34 and CD45 are hematopoietic markers (A); both CD44 and CD90 are mesenchymal markers (B).

Flow cytometry was used to confirm the type of cultured cells by detecting antigenic markers. The antigens that were used are CD34 and CD45 as hematopoietic cell markers (negative markers) and CD44 and CD90 as mesenchymal cell markers (positive markers). The results showed that 1.86% of cells had CD34 marker and 0.139% of cells had CD45 marker (Figure 3A). Additionally, 100% of cells had CD44 marker, and 99.7% of cells had CD90 marker (Figure 3B).

### 3.4 Cell Count in the Ventral and Dorsal Parts of the Striatum

**FIGURE 4:**
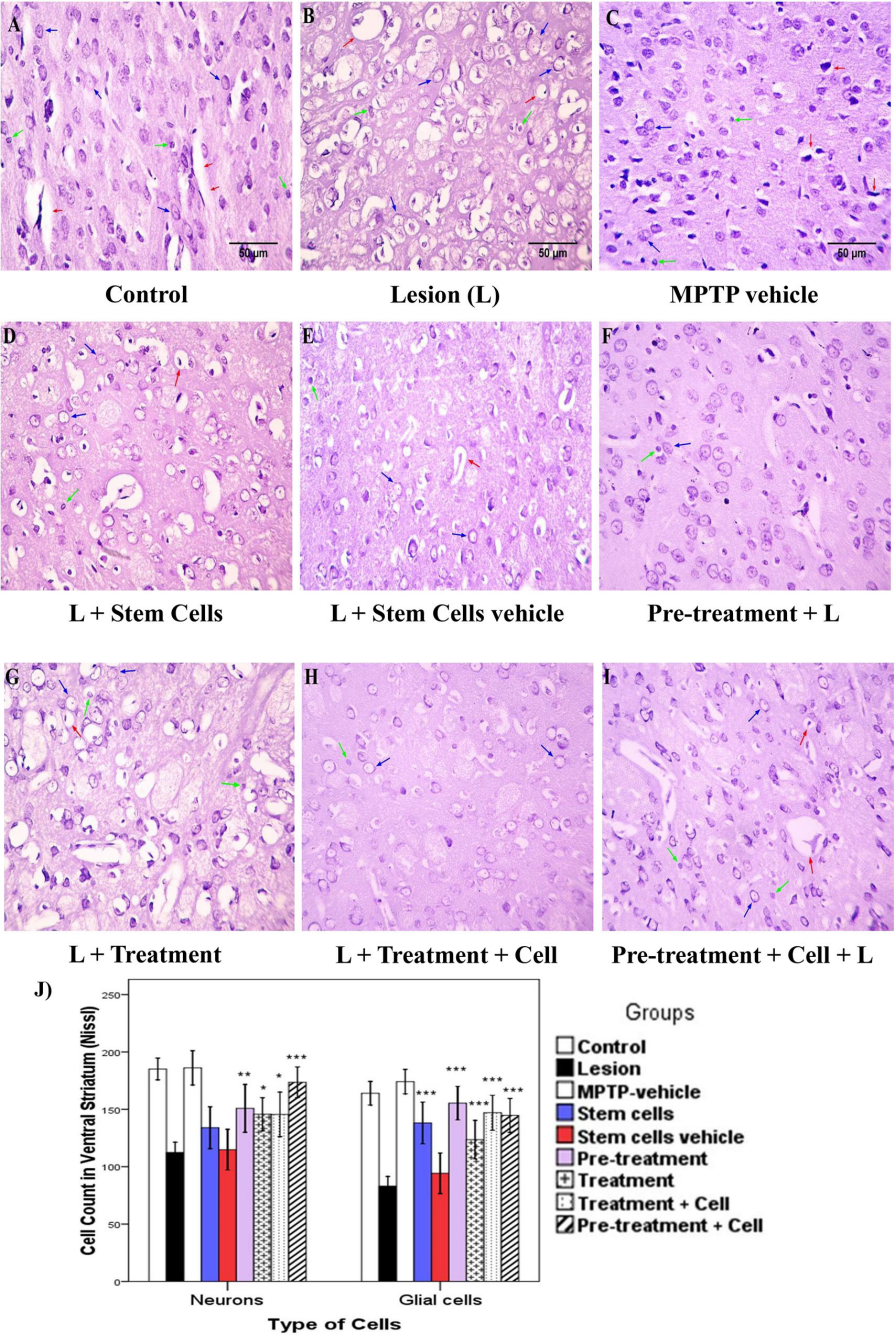
Ventral part of the striatum (Nissl stain). Blue, green, and red arrows indicate neurons, glial cells, and blood vessels, respectively: (A) control; (B) lesion; (C) MPTP vehicle; (D) stem cells; (E) stem cells vehicle (culture media); (F) pretreatment; (G) treatment; (H) treatment plus stem cells; (I) pretreatment plus stem cells. Magnification ×400; scale bar 50 µm. (J) Data are shown as the mean ± SD of neurons and glial cells in the ventral striatum and were analyzed using one‐way ANOVA followed by the Tukey post hoc test. MPTP, 1‐methyl‐4‐phenyl‐1,2,3,6‐tetrahydropyridine. *p < 0.05; **p < 0.01; ***p < 0.001.

In the ventral part of the striatum, neurons and glial cells were counted separately using Nissl staining. For this purpose, properties such as nucleus size and nucleus condensation (euchromatin or heterochromatin) were used. The neuron counts in different groups are as follows: Comparison of control and MPTP vehicle groups with the lesion group showed a significant difference (*p *< 0.001). The stem cells and stem cells vehicle groups did not significantly differ from the lesion group (*p *> 0.05). The pretreatment (*p *= 0.003), treatment (*p *= 0.016), treatment plus cell (*p *= 0.017), and pretreatment plus cell (*p *< 0.001) groups all showed significant differences compared to the lesion group. Underlying the evaluation of glial cells count, results are as follows: Stem cells vehicle group did not show a significant difference with the lesion group (*p *> 0.05). The stem cells, pretreatment, treatment, treatment plus cell, and pretreatment plus cell groups all showed a significant difference (*p *< 0.001) compared to the lesion group (Figure 4).

**FIGURE 5:**
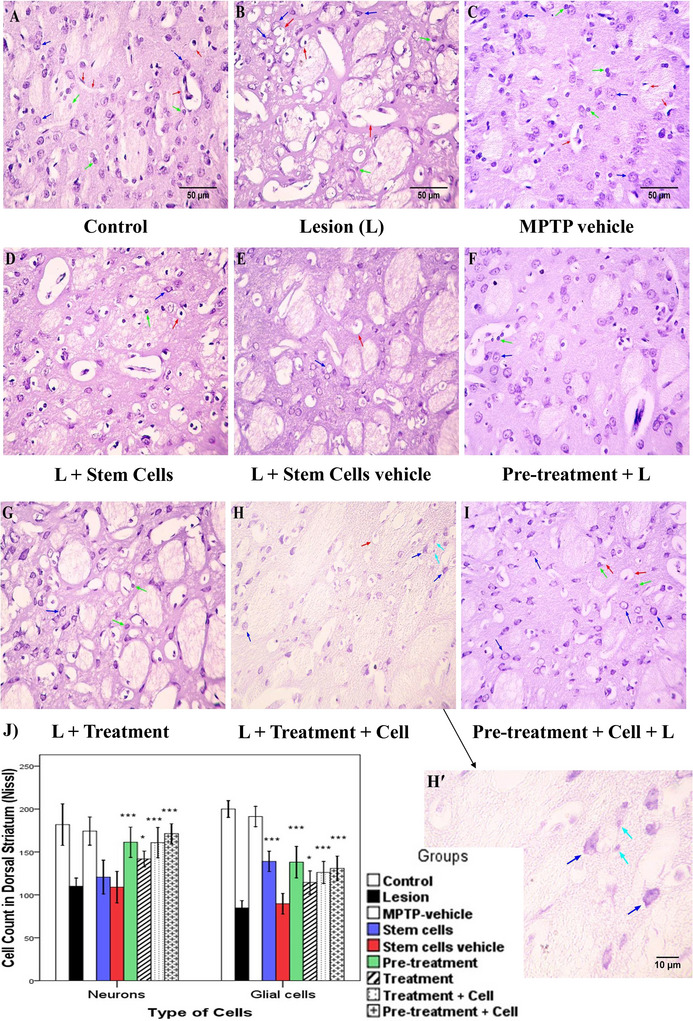
Dorsal part of the striatum (Nissl stain). Blue, green, and red arrows indicate neurons, glial cells, and blood vessels, respectively: (A) control; (B) lesion; (C) MPTP vehicle; (D) stem cells; (E) stem cells vehicle (culture media); (F) pretreatment; (G) treatment; (H) treatment plus stem cells; (I) pretreatment plus stem cells. Magnifications ×400 (A–I) and ×1000 (H′); scale bars 10 and 50 µm.(J) Data are given as the mean ± SD of neurons and glial cells in the dorsal striatum and were analyzed using one‐way ANOVA followed by the Tukey post hoc test. MPTP, 1‐methyl‐4‐phenyl‐1,2,3,6‐tetrahydropyridine. *p < 0.05; ***p < 0.001.

For the dorsal part, representative images are shown in Figure 5. Concerning neurons, the stem cells and stem cells vehicle groups did not differ significantly from the lesion group (*p *> 0.05). However, the treatment (*p *= 0.041), pretreatment, treatment plus cell, and pretreatment plus cell (*p *< 0.001) groups showed significant differences from the lesion group. Regarding the glial cells count, stem cells vehicle group did not show a significant difference with the lesion group (*p *> 0.05). The stem cells, pretreatment, treatment plus cell, and pretreatment plus cell groups (*p *< 0.001), as well as the treatment group (*p *= 0.005), showed significant differences compared to the lesion group (Figure 5). The numerical values are presented in Table 1.

**TABLE 1:** Behavioral evaluation of rats and histological evaluation of the ventral and dorsal striatum (Nissl staining).

Groups	Pole test	Ventral striatum (neurons)	Ventral striatum (glial cells)	Dorsal striatum (neurons)	Dorsal striatum (glial cells)
Control	5.1 ± 0.63	185.1 ± 10.25	164 ± 11.13	181.8 ± 26.15	200 ± 10.52
Lesion	2.1 ± 0.25	112.4 ± 9.69	83.1 ± 8.98	110 ± 10.29	84.7 ± 9.26
MPTP‐vehicle	4.6 ± 0.70	186.1 ± 16.12	174.1 ± 11.51	174.2 ± 17.74	191.2 ± 12.8
Stem cells	2.4 ± 0.56	134 ± 19.74	138.1 ± 19.6***	120.7 ± 21.26	139 ± 12.83***
Stem cells‐vehicle	2 ± 0.66	114.8 ± 19.23	94.2 ± 19.12	109 ± 19.74	89.7 ± 12.93
Pretreatment	4.2 ± 0.63***	150.8 ± 22.5**	155.4 ± 15.74***	161.2 ± 19.07***	138.1 ± 19.79***
Treatment	2.5 ± 0.74	145.7 ± 15.62*	123.5 ± 18.17***	141.8 ± 9.89*	114.4 ± 14.59*
Treatment + cell	2.5 ± 0.43	145.5 ± 20.99*	147 ± 16.49***	160.8 ± 19.07***	126.1 ± 13.82***
Pretreatment + cell	3.4 ± 0.53**	173.5 ± 14.44***	144.7 ± 15.95***	171.4 ± 12.32***	130.8 ± 15.46***

*Note*: Values are expressed as mean ± SD and were analyzed using one‐way ANOVA followed by the Tukey post hoc test (*n* = 6–7).

Abbreviation: MPTP, 1‐methyl‐4‐phenyl‐1,2,3,6‐tetrahydropyridine.

*p < 0.05.

**p < 0.01.

***p < 0.001 versus lesion.

### 3.5 Cell Count in the SNC Area

**FIGURE 6:**
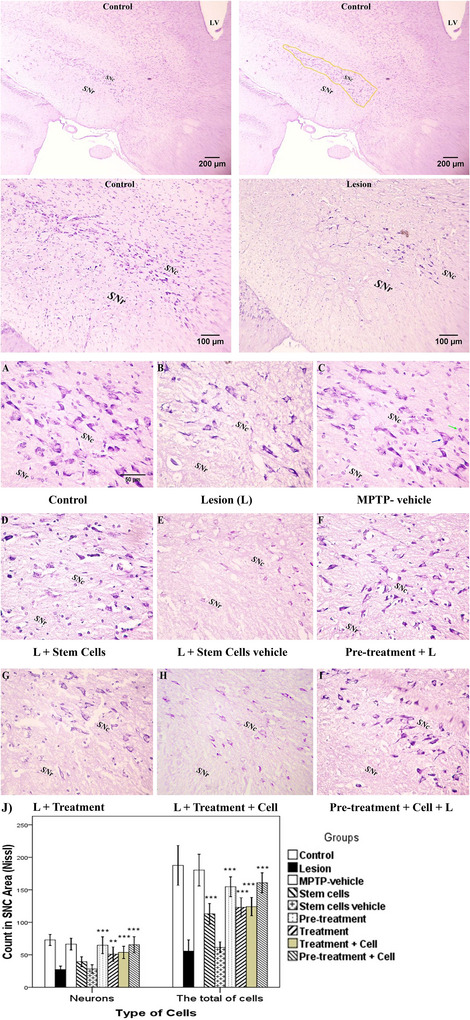
The evaluation of neurons and glial cells in the substantia nigra pars compacta (SNC). Blue and green arrows indicate neurons and glial cells, respectively: (A) control; (B) lesion; (C) MPTP vehicle; (D) stem cells; (E) stem cells vehicle (culture media); (F) pretreatment; (G) treatment; (H) treatment plus stem cells; (I) pretreatment plus stem cells. Magnifications ×40, ×100, and ×400; scale bars 50, 100, and 200 µm; Nissl stain. (J) Data are expressed as the mean ± SD of neurons and total of cells in the SNC area and were analyzed using one‐way ANOVA followed by the Tukey post hoc test. The total of cells, that is, the sum of neurons and glial cells. MPTP, 1‐methyl‐4‐phenyl‐1,2,3,6‐tetrahydropyridine. **p < 0.01; ***p < 0.001.

**FIGURE 7:**
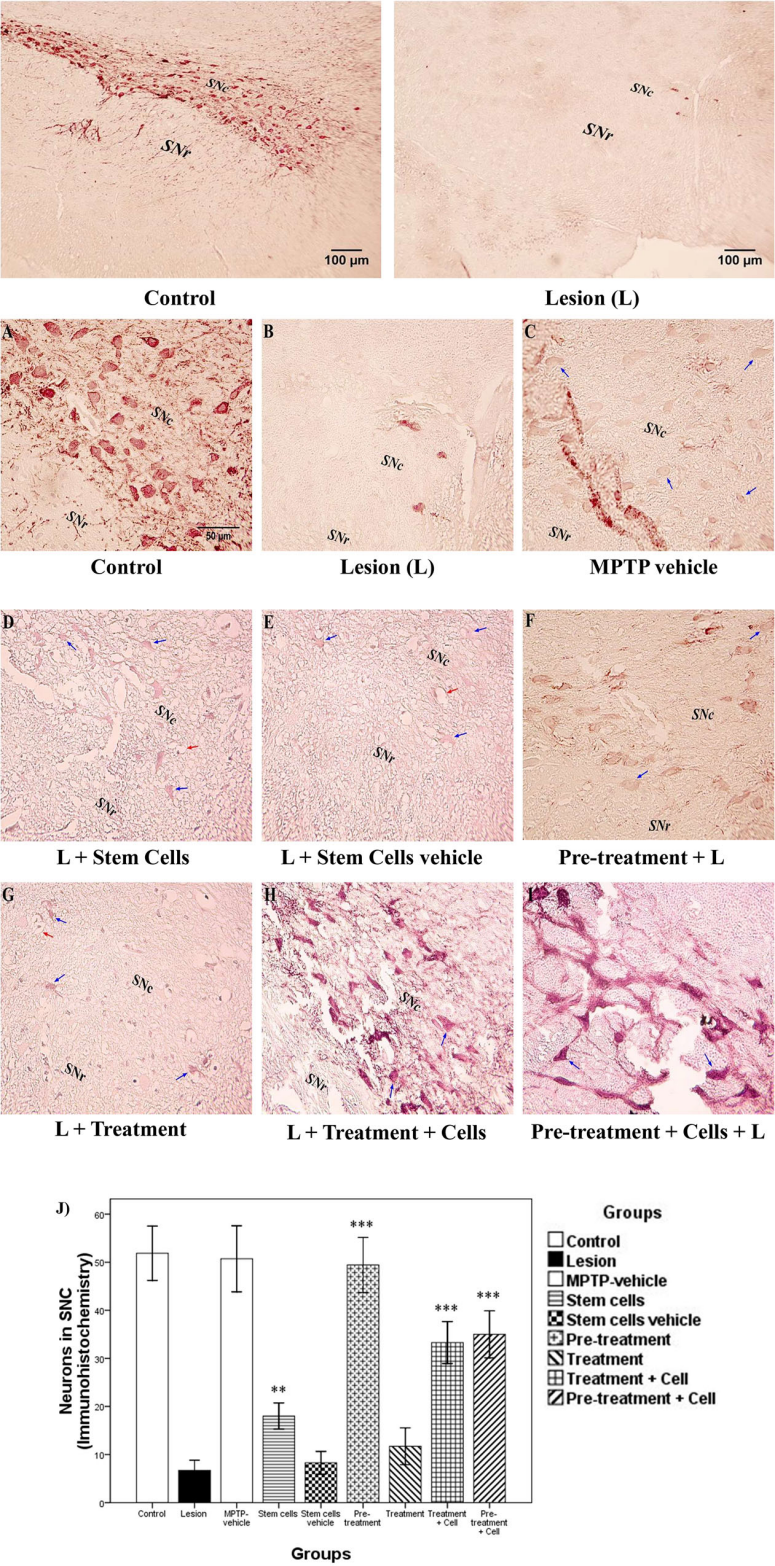
The evaluation of dopaminergic neurons in the substantia nigra pars compacta (SNC). Blue and red arrows indicate dopaminergic neurons and blood vessels, respectively: (A) control; (B) lesion; (C) MPTP vehicle; (D) stem cells; (E) stem cells vehicle (culture medium); (F) pretreatment; (G) treatment; (H) treatment plus stem cells; (I) pretreatment plus stem cells. Magnifications ×100 and ×400; scale bars 50 and 100 µm; immunohistochemistry (TH marker). (J) Data are represented as the mean ± SD of dopaminergic neurons in the SNC area and were analyzed using one‐way ANOVA followed by the Tukey post hoc test. MPTP, 1‐methyl‐4‐phenyl‐1,2,3,6‐tetrahydropyridine. **p < 0.01; ***p < 0.001.

Photos are shown in Figure 6. Concerning neurons, the stem cells and stem cells vehicle groups did not significantly differ from the lesion group (*p *> 0.05). The pretreatment, treatment plus cell, and pretreatment plus cell (*p *< 0.001) groups, as well as the treatment group (*p *= 0.002), showed significant differences from the lesion group. Regarding the total number of cells (neurons and glial cells), there were no significant differences between the stem cells vehicle group and the lesion group, which is expected (*p *> 0.05). However, the stem cells, treatment, pretreatment, treatment plus cell, and pretreatment plus cell groups (*p *< 0.001) all showed significant differences (Figure 6). On the underlying dopaminergic neurons, the treatment group did not significantly differ from the lesion group (*p *> 0.05), whereas the stem cells group (*p *= 0.002), pretreatment, treatment plus cell, and pretreatment plus cell groups (*p *< 0.001) showed significant differences compared to the lesion group (Figure 7).

### 3.6 Determining SOD and GPX Activities

**FIGURE 8:**
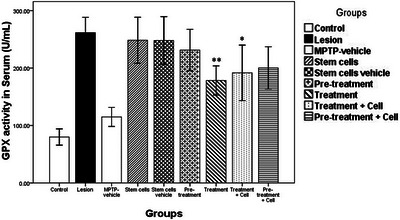
Data are represented as the mean ± SD of GPX activity in serum (U/mL) and were analyzed using one‐way ANOVA followed by the Tukey post hoc test. GPX, glutathione peroxidase; MPTP, 1‐methyl‐4‐phenyl‐1,2,3,6‐tetrahydropyridine. *p < 0.05; **p < 0.01.

The activities of CuZn‐SOD and Mn‐SOD enzymes were reported as total SOD activity, following the kit instructions. The assay range for SOD activity was 5–100 U/mL, and for GPX, it was 20–500 U/mL. For the SOD enzyme, no significant differences were observed between any groups and the lesion group (*p *> 0.05; data not shown). Regarding the GPX enzyme, the stem cells, stem cells vehicle, pretreatment, and pretreatment plus cell groups did not show significant differences from the lesion group (*p *> 0.05). However, the treatment group (*p *= 0.002) and treatment plus cell group (*p *= 0.018) showed significant differences compared to the lesion group (Figure 8). Data are expressed in Table 2.

**TABLE 2:** Histological evaluation on the SNC area (Nissl and Immunohistochemistry staining); activity evaluation of the oxidative stress enzymes within serum (U/mL).

Groups	SNC (neurons)	SNC (total of the cells)	SNC (IHC)	SOD activity	GPX activity
Control	72.8 ± 9.22	187.5 ± 33	51.8 ± 6.12	45.6 ± 1.86	79.9 ± 15.31
Lesion	27.2 ± 5.73	55.5 ± 18.75	6.7 ± 2.28	48.4 ± 2.48	261.6 ± 28.86
MPTP‐vehicle	66.4 ± 9.69	180.2 ± 26.34	50.7 ± 7.43	40.9 ± 3.33	114.9 ± 17.94
Stem cells	39.4 ± 8.1	113 ± 17.08***	18 ± 2.94	44.8 ± 4.20	248.4 ± 43.30
Stem cells‐vehicle	28.2 ± 6.82	61.4 ± 9.01	8.2 ± 2.56	49 ± 7.30	248 ± 44.86
Pretreatment	64.7 ± 13.97***	154.7 ± 16.43***	49.4 ± 6.18***	44.1 ± 5.85	231.3 ± 38.87
Treatment	51 ± 11.93**	123 ± 15.86***	11.7 ± 4.15	48.2 ± 7.63	178.4 ± 27.44**
Treatment + cell	53.7 ± 10.21***	124.1 ± 15.12***	33.2 ± 4.71***	46 ± 3.24	191.6 ± 52.23*
Pretreatment + cell	65.4 ± 13.37***	160.8 ± 16.4***	35 ± 5.29***	48.9 ± 3.08	200.2 ± 39.86

*Note*: Total of the cells, that is, the sum of neurons and glial cells; superoxide dismutase (SOD); glutathione peroxidase (GPX). Data are given as mean ± SD and were analyzed using one‐way ANOVA followed by the Tukey post hoc test (*n *= 6–7 for histology and *n *= 5–7 for serology).

Abbreviations: IHC, immunohistochemistry; MPTP, 1‐methyl‐4‐phenyl‐1,2,3,6‐tetrahydropyridine; SNC, substantia nigra pars compacta.

*p < 0.05.

**p < 0.01.

***p < 0.001 versus lesion.

## 4 Discussion

This research, that is, examining the effect of St. John's wort extract on PD, has not been done so far, and we tested it for the first time on a model of PD, but among the ingredients of this extract, quercetin and kaempferol have been tested on Parkinson's model. We found that St. John's wort has a strong protective effect against Parkinson's progression, nearly as effective as when it is combined with stem cell treatment. However, stem cells alone—without a targeted genetic program—are limited in function and must be guided toward dopaminergic neurons through gene regulation, particularly during differentiation. Additionally, this herbal medicine as well as its ingredients have amazing abilities that must be identified, and its special ingredients should be purified and examined more. For instance, kaempferol appears particularly promising and has been tested previously in a rotenone‐induced Parkinson's model. In the present study, IHC for the TH enzyme demonstrated that St. John's wort is highly effective at preventing dopaminergic neuron loss and may benefit elderly people or those in the early stages of PD. On the basis of the study findings, the treatment plus stem cells and pretreatment plus stem cells groups showed similar recovery effects. In my own view, this similarity may be due to compounds like quercetin and kaempferol in the extract, which have previously demonstrated activity on the nervous system. In the treatment plus stem cells and pretreatment plus stem cells groups, some DiI‐stained cells were observed in the injured striatum, which may indicate stem cell migration and guidance mediated by St. John's wort ingredients, but limited stem cell migration to the substantia nigra suggests the need for improving cell delivery methods or genetic modifications. No such evidence was found in the stem cells group like this. It has been proven the effectiveness of quercetin on the striatum, which could be a valid reason for the efficacy of SHP. Studies on PD have shown that kaempferol possesses antiapoptotic and antioxidant properties, protecting mitochondrial transmembrane transmission function and oxygen consumption while reducing ROS and carbonyl levels (Filomeni et al. 2012; Haleagrahara et al. 2011). Research has confirmed that kaempferol reduces intracellular ROS and apoptosis by over 50%, while also upregulating mRNA expression of the TH enzyme and its associated proteins (Pan et al. 2020). However, little research has been conducted on the other active ingredients of this plant and their effects on PD—aside from kaempferol—which limits our ability to analyze their impact on the striatum. So, it could be a reason for keeping on the study of extract's effectiveness. Studies of the striatum in Huntington's disease models have shown that secreted factors from grafted mesenchymal and neuronal stem cells can support host cells in the damaged striatum. These factors can activate signaling pathways and enhance both migration and proliferation of stem cells. It has been demonstrated that mesenchymal stem cells transplantation in Parkinson's animal models improves neuronal function in the striatum (Bantubungi et al. 2008; Fu et al. 2013).

On the basis of the Nissl staining and quantitative analysis of neurons and glial cells in the SNC area, both the extract and cell therapy were effective in recovery or maintenance. However, the most notable improvements occurred in the combined therapy groups (G8 and G9), suggesting a synergistic effect of stem cells and the extract. Pretreatment alone also demonstrated its own benefits. This indicates that St. John's wort provides strong protection against pathogenic factors. It is important to identify which active ingredient is the most responsible for the observed changes in this brain region. One of these ingredients is rutin that prevents nerve cells from apoptosis through stopping the expression of BAX pre‐apoptotic gene and inhibiting the activity of Caspase‐3 (Na et al. 2014). Research has shown that rutin attenuates the expression of Park2, Park5, Park7, Caspase‐3, and Caspase‐5 genes and helps to upregulate TH gene. Additionally, rutin and its isomers contribute to the expression of anti‐apoptotic genes such as Opa1 and NSF, which support the preservation of dopaminergic cells (Magalingam et al. 2015). Parallel to that, mesenchymal stem cells also in its own right protect TH‐positive neurons in SNC and do it in a variety of ways (Suzuki et al. 2015). For example, they secrete neuroprotective and neurotrophic factors such as basic fibroblast growth factor (bFGF), GDNF, and ciliary neurotrophic factor (CNTF) and may also differentiate into dopaminergic neurons or glial cells, thereby helping protect SNC neurons from the neurotoxic damage (Blandini et al. 2010; Yang et al. 2014).

The *N*‐methyl‐d‐aspartate (NMDA) receptor is one of the receptors implicated in PD, although research on it remains incomplete. It is an ion channel for positively charged ions (cations) and one of the most abundant membrane receptors in neurons. Upon activation, it allows calcium and sodium ions to enter the cell and potassium to exit, playing a crucial role in synapse formation and memory function. The receptor is activated through two binding sites—one for glutamate and another for glycine or serine—and can be inhibited via an allosteric site by binding endogenous or exogenous molecules (C.‐H. Lee et al. 2014). For example, hyperforin, an active compound in St. John's wort, appears to act as an exogenous antagonist molecule for the NMDA receptor, thereby inhibiting its activity (Kumar et al. 2006). Inhibition of the NMDA receptor may slow disease progression by delaying the degeneration of dopaminergic neurons. Thus, antagonists of NMDA receptor might be able to reverse motor symptoms in preclinical PD models and be useful in preventing the neurodegeneration (Johnson et al. 2009). On the other hand, this inhibition of activity must be accompanied by proper and constructive regulation. Let us not forget that the NMDA receptor is the most abundant receptor on nerve cells so that its activity is certainly essential, as well as its complete and absolute inhibition can have harmful effects and lead to symptoms. Therefore, proper and rational regulation of the activity of this receptor must be identified and applied to help maintain the body's natural balance and homeostasis. Some studies found that pretreatment with quercetin, rutin, and okra extract prevented the reduction of NMDA receptor expression and restored BrdU‐immunoreactivity in the dentate gyrus of the brain. These findings suggest that quercetin, rutin, and okra extract inverted cognitive deficits, including impaired cell proliferation of the dentate gyrus, and preserved the CA3 region against morphological changes in dexamethasone‐treated mice. Rutin was investigated on PD as in vitro (*SH‐SY5Y* human neurons cell line), and it has also been tested on schizophrenia into preventive treatment (Enogieru et al. 2021; Hai et al. 2025; Oshodi et al. 2021; Saputri et al. 2025; Tongjaroenbuangam et al. 2011). Continuing with the hyperforin content, hyperforin may also influence cellular processes and can interact with several signaling pathways that are crucial for stem cell maintenance and differentiation. A study on diabetes reported that St. John's wort and hyperforin remarkably protect pancreatic beta cells (β‐cells) in vitro against the deleterious effects of immune and inflammatory cytokines by inhibiting multiple phosphorylation steps of cytokine signaling (Novelli et al. 2016). In this context, that is, specifically its influence on stem cells, St. John's wort may affect the migration and differentiation of certain types of stem cells. For example, it may promote differentiation of mesenchymal stem cells into adipocytes (fat cells), while inhibiting osteoblastic differentiation. Therefore, in vitro more investigation of St. John's wort or its individual compounds on stem cells or neural cells is recommended (Casado‐Díaz et al. 2016). The results of another study demonstrated that **hyperforin**, one of the potent constituents of H. perforatum, ameliorates microglial activation and white matter lesions, ultimately improving cognitive function in vascular cognitive impairment (VCI) mice through the VEGFR2/SRC pathway. The impact of hyperforin on this pathway was examined using western blot analysis. The results indicated increased expression levels of VEGFR2, p‐SRC/SRC, and VEGFA in BV2 microglial cells subjected to oxygen–glucose deprivation/reperfusion (OGD/R). Hyperforin significantly attenuated microglial M1 polarization by modulating the VEGFR2/SRC pathway in OGD/R‐induced BV2 microglia. Thus, hyperforin ameliorates neuroinflammation and white matter lesions by regulating microglial VEGFR2/SRC signaling in VCI mice (Gao et al. 2024). In PD research, it was shown that **rutin** plays an important role in improving histological, biochemical, and behavioral parameters following haloperidol administration in rats, thereby confirming its protective effects (Sharma et al. 2016). Furthermore, rutin has been identified as a promising drug candidate for Alzheimer's disease due to its combinatorial targeting of tau and Aβ (Sun et al. 2021). The binding mechanism of rutin in Alzheimer's models was investigated at the atomic level using molecular docking and extensive 200‐ns molecular dynamics simulations. Molecular docking revealed that rutin binds within the binding pocket of human transferrin, whereas molecular dynamics simulations demonstrated that this interaction does not induce significant structural changes or disrupt the native packing of transferrin (Shamsi et al. 2023).

There is growing interest in the potential of St. John's wort to promote neurogenesis, particularly due to its mood‐enhancing properties and its well‐documented role in treating depression. This may involve interactions with neural stem cells and their differentiation into neurons, although more research is needed in this area. Overall, analysis of cell surface markers (CD44 and CD90) confirmed robust BMSCs growth, and DiI tracking showed that these pluripotent stem cells reached to the striatum, likely via cerebrospinal fluid flow and facilitated by St. John's wort administration. In other words, the presence of these cells within striatum indicates that stem cells can immigrate into the central nervous system through CSF flow, aided by neuroprotective and neurotrophic factors (Ye et al. 2018). The limited arrival and establishment of stem cells in the SNC via cerebrospinal fluid flow could be due to two reasons: (1) The lack of proximity to the lateral ventricles. (2) Either stem cells do not have the ability to add to the SNC tissue because of their intact and primary situation, or it happens very weakly unless they undergo genetic modification. The findings have shown that the wingless‐type MMTV integration site (Wnt) pathway undertakes the main role in midbrain dopaminergic neurogenesis. Overall, both Wnt/β‐catenin‐dependent (canonical Wnt) and Wnt/β‐catenin‐independent (non‐canonical Wnt) cascades of Wnt signaling are involved in the healthy function and regulation of the adult brain. The Wnt/β‐catenin (canonical Wnt) signaling is an evolutionarily preserved pathway that has a crucial role not only in normal embryonic development but also in the preservation of adult brain tissue homeostasis as well. If these pathways are deregulated for so long, it will probably lead to compromise neural activity. On the contrary, multiple recent evidence illustrated that the upregulation of the Wnt/β‐catenin pathway is able to reactivate neurogenesis and provoke the inherent self‐repair capacities in the damaged brain and could also help stem cells differentiate into dopaminergic neurons (Gong et al. 2020; Marchetti et al. 2020; Westphal et al. 2022; Zhang et al. 2015).

The SOD and GPX enzymes often work in tandem within cellular antioxidant defense mechanisms. SOD catalyzes superoxide (O_2_^−^) into hydrogen peroxide (H_2_O_2_) and molecular oxygen (O_2_). Following this, GPX catalyzes the reduction of hydrogen peroxide to water (H_2_O) using glutathione (GSH) as a reducing agent. This sequential action helps manage the levels of superoxide and hydrogen peroxide in cells. However, research to date has yielded mixed results regarding oxidative stress enzymes and their role in PD. In a study, in vitro research suggests that MPTP‐induced toxicity in dopaminergic *PC12* cell cultures does not involve the production of oxygen free radicals; rather, it may be due to disruption of energy metabolism. In other words, toxic effects of MPTP and MPP+ in *PC12* cells are independent of ROS formation (Fonck and Baudry 2001). Another study showed that oxidative stress plays an important role in various neurodegenerative diseases particularly PD. In order to clarify this issue, PD models are generated by MPTP intramuscular injection in eight rhesus monkeys. During PD pathogenesis progression, the activities of some major antioxidant enzymes, such as serum SOD, GPX, and glutathione‐s‐transferase (GST), were measured and showed continuously decrease (L. Li et al. 2017). However, examining oxidative stress enzymes is not without merit and allows us to understand whether St. John's wort extract and its compounds take a step towards strengthening the oxidative stress system. St. John's wort possesses both anti‐inflammatory and antioxidant properties (Orhan et al. 2014; Paulke et al. 2008), which were evident in this study. On the basis of our findings, SOD activity did not significantly differ across groups, including lesion and control, suggesting that its substrate—superoxide (O_2_^−^)—was likely not overproduced, thus not requiring increased SOD activity. In other words, levels of superoxide in different groups were almost the same which led to a uniform level of SOD enzyme in different groups. However, GPX activity varied significantly between groups, indicating differing levels of its substrate, hydrogen peroxide (H_2_O_2_). Given that rutin can inhibit monoamine oxidase (MAO)—which typically reduces GPX activity—its presence in the extract may account for the observed increase in GPX levels (Dimpfel 2009).

The long‐term effects and safety of the treatment with St. John's wort extract have been studied, and no specific harm has been reported so far, because St. John's wort is one of the most widely used herbal antidepressants in the Europe and America, but no basic research and clinical trials have been conducted on combination therapy or simultaneous use of the St. John's wort extract and receiving stem cells which can be cited them. Overall, variant studies have been performed regarding long‐term effects and safety of the remedy with stem cells and also combined therapy with other chemical or herbal drugs. In a double‐blind study at 49 hospitals in Europe, including 212 patients with Crohn's disease and treatment‐refractory, complex perianal fistulas, patients were randomly assigned (1:1) to groups given a single local injection of 120 million *Cx601* cells or placebo (control), in addition to the standard of care. The result of this clinical trial (phase 3) of patients with Crohn's disease and treatment‐refractory complex perianal fistulas showed *Cx601* cells to be safe and effective in closing external openings, compared with placebo, after 1 year (Panés et al. 2018).

Ultimately, understanding the relationship between St. John's wort and stem cells—particularly in terms of their deployment and differentiation—may hold potential therapeutic implications, especially in regenerative medicine and the treatment of neurodegenerative diseases. Beyond its traditional use for depression and burns, which remains relatively common, the clinical applications of St. John's wort in these emerging areas are still largely speculative. Therefore, studies involving different dosages and long‐term monitoring are recommended to further evaluate potential side effects and therapeutic efficacy.

## 5 Conclusion

The study found that both *H. perforatum* extract and BMSCs can help to preserve dopaminergic neurons and glial cells. The combined treatment—especially when used as pretreatment—produced a strong synergistic effect. Additionally, medicinal pretreatment alone showed results nearly comparable to the combination group. Evidence suggests that cell therapy has a stronger effect on preserving glial cells, possibly due to their origin and molecular signaling within the brain, or because these stem cells, without genetic programming, may not differentiate effectively into neurons. Overall, St. John's wort is a unique medicinal plant and natural protector that supports the survival of both neurons and glial cells against destructive environmental factors and pathogens, offering potential therapeutic value for PD. Further research is recommended on individual compounds such as kaempferol, hyperforin, and rutin, exploring varying dosages and the use of gene therapy to examine their effects on the expression of dopamine synthesis enzymes.

## Author Contributions

**Hamed Farzadmanesh**: conceptualization, visualization, methodology, formal analysis, investigation, writing–original draft. **Hamidreza Sameni**: conceptualization, visualization, methodology. **Ali Ghanbari**: conceptualization, visualization, methodology. **Abbas Ali Vafaei**: conceptualization, visualization, methodology. **Ali Khaleghian**: conceptualization, visualization, methodology. **Majid Mirmohammadkhani**: formal analysis, investigation. **Laya Ghahari**: writing–review and editing. **Afsaneh Shokri**: writing–review and editing. **Manouchehr Safari**: conceptualization, visualization, methodology, writing–review and editing, supervision.

## Funding

Funding acquisition and resources of this study were financed by SEMUMS (Grant IR.SEMUMS.REC.1397.188).

## Conflicts of Interest

The authors declare no conflicts of interest.

## Data Availability

Data will be presented upon request.
